# Elamipretide (SS-31) Ameliorates Isoflurane-Induced Long-Term Impairments of Mitochondrial Morphogenesis and Cognition in Developing Rats

**DOI:** 10.3389/fncel.2017.00119

**Published:** 2017-04-25

**Authors:** Jing Wu, Shuangying Hao, Xiao-Ru Sun, Hui Zhang, Huihui Li, Hongting Zhao, Mu-Huo Ji, Jian-Jun Yang, Kuanyu Li

**Affiliations:** ^1^Jiangsu Key Laboratory of Molecular Medicine, Medical School of Nanjing UniversityNanjing, China; ^2^Medical School of Henan Polytechnic UniversityJiaozuo, China; ^3^Department of Anesthesiology, Zhongda Hospital, School of Medicine, Southeast UniversityNanjing, China

**Keywords:** elamipretide, general anesthesia, antioxidant, mitochondrial morphology, cognition

## Abstract

Mitochondria are supposed to be involved in the early pathogenesis of general anesthesia (GA)-induced neurotoxicity and long-term cognitive deficits in developing brains. However, effective pharmacologic agents targeted on mitochondria during GA exposure are lacking. This study explores the protective effects of mitochondrion-targeted antioxidant elamipretide (SS-31) on mitochondrial morphogenesis and cognition in developing rats exposed to isoflurane. Rat pups at postnatal day (PND) 7 were exposed to 1.5% isoflurane for 6 h following intraperitoneal administration of elamipretide or vehicle with 30 min interval. The hippocampus was immediately removed for biochemical assays. Histopathological studies were conducted at PND 21, and behavioral tests were performed at PND 40 or 60. We found that early exposure to isoflurane caused remarkable reactive oxygen species (ROS) accumulation, mitochondrial deformation and neuronal apoptosis in hippocampus. The injury occurrence ultimately gave rise to long-term cognitive deficits in developing rats. Interestingly, pretreatment with elamipretide not only provided protective effect against oxidative stress and mitochondrial damages, but also attenuated isoflurane-induced cognitive deficits. Our data support the notion that mitochondrial damage is an early and long lasting event of GA-induced injury and suggest that elamipretide might have clinically therapeutic benefits for pediatric patients undertaking GA.

## Introduction

Thousands of infants and children require prolonged periods of anesthesia when complicated surgery is needed each year. A large number of preclinical studies have raised concern about the potential of neurotoxicity and long-term cognitive impairments caused by general anesthetics in developing brains (Ing et al., 2014a; Lin et al., 2014; Rappaport et al., 2015; Walters and Paule, 2017). Due to the increasing demand for pediatric patients to have anesthesia during surgery, recent research efforts are focusing on deciphering the earliest cellular targets and mechanistic pathways of general anesthesia (GA)-induced developmental neurotoxicity so that strategies for effective ameliorating or preventing the adverse effects can be devised (Boscolo et al., 2012, 2013a; Jevtovic-Todorovic et al., 2012; Noguchi et al., 2016).

Impaired mitochondrial function and morphology are centrally involved in the neurotoxicity and play key pathogenic roles in GA-induced cognitive deficits (Sanchez et al., 2011; Boscolo et al., 2012, 2013a,b). The mitochondrial abnormalities exist in vulnerable brain regions evidenced by increased reactive oxygen species (ROS) generation and lipid peroxidation, mitochondrial enlargement, and impaired structural integrity (Sanchez et al., 2011; Boscolo et al., 2012, 2013a,b). However, efforts to directly target mitochondria to limit ROS and oxidative stress and modify mitochondrial function during GA exposure are still in progress because of the absence of effective pharmacological agents. Elamipretide (SS-31, D-Arg-dimethylTyr-Lys-Phe-NH_2_) is a novel mitochondrion-targeted antioxidant. It has an alternating aromatic-cationic structure that allows it to freely cross the blood-brain barrier and cell membrane, then concentrate >1000 fold in the mitochondrial inner membrane independently of mitochondrial membrane potential. It has been demonstrated that elamipretide inhibits ROS generation and lipid peroxidation in many processes both *in vitro* and *in vivo* (Zhao et al., 2005; Szeto, 2008; Min et al., 2011; Hao et al., 2015; Yin et al., 2016). Our previous studies provided evidences that elamipretide protected mitochondrial function by reducing ROS generation, maintaining adenosine triphosphate (ATP) production and mitochondrial membrane potential, and inhibiting opening of mitochondrial permeability transition pore in aged mice receiving isoflurane anesthesia (Wu et al., 2015a, 2016). However, the efficacy of elamipretide has yet to be studied in the developing rats.

In the present study, we sought to examine the antioxidative and mitochondrial protective effects of elamipretide in rats during the critical stage of brain development. We administered elamipretide and isoflurane anesthesia to rat pups at postnatal day (PND) 7 and discovered that elamipretide treatment ameliorated isoflurane-induced oxidative stress and long-term impairments of mitochondrial morphogenesis and cognition in developing rats.

## Materials and Methods

### Animals

Sprague-Dawley rat pups at PND 7 were used in the present study. All experimental procedures and protocols were reviewed and approved by the Animal Investigation Ethics Committee of Nanjing University and were performed in accordance with the Guidelines for the Care and Use of Laboratory Animals from the National Institutes of Health, USA. The pups were housed in a room maintained under standard environmental condition (temperature 22–24°C, a 12 h light/dark cycle, and 50 ± 10% humidity) with their mothers till PND 20. At PND 21, the pups were weaned and housed 4–5 per cage in standard condition.

### Experimental Protocols

Ninety-six rat pups at PND 7 of both sexes were randomly assigned to one of the following four treatment protocols (*n* = 24/group): control, control + elamipretide, isoflurane and isoflurane + elamipretide. Elamipretide (5 mg/kg, synthesized in China Peptides Co., Ltd., Shanghai, China) or phosphate-buffered saline (PBS) was intraperitoneally administered to the pups with a volume of 0.4 ml/kg 30 min before gas inhalation. The dose of elamipretide was chosen according to our previous studies (Wu et al., 2015a,b, 2016). Anesthesia was performed based on previous optimization, in which 6 h isoflurane anesthesia can induce developmental neurotoxicity (Boscolo et al., 2012, 2013a; Wang et al., 2014; Xu et al., 2015). Anesthesia was induced by placing the pups in an anesthetizing chamber prefilled with 1.8% isoflurane plus 30% oxygen (O_2_) for 10 min and then changed to 1.5% isoflurane plus 30% O_2_ for 350 min. For control experiments, 30% O_2_ was delivered for 6 h at the same flow rate. The composition of the chamber gas was continuously monitored using a DatexTM infrared analyzer (Capnomac, Helsinki, Finland). Rats were kept normothermic throughout the experiment.

After the administration of anesthesia, rats were randomly divided into three subgroups. Six rats in subgroup 1 were sacrificed immediately postanesthesia (at PND 7), and the hippocampi were rapidly removed for biochemical studies. Six rats in subgroup 2 were sacrificed at PND 21 and the brain of each rat was cut into two halves for histopathological studies, and 12 rats in subgroup 3 were used for behavioral studies at PND 40 or 60. Efforts were made to minimize the number of animals used in this study.

### ROS Level, Superoxide Dismutase (SOD) Activity and Malondialdehyde (MDA) Content Assays

Rats (*n* = 6 for each group) were sacrificed immediately postanesthesia (at PND 7), and the hippocampi were removed quickly. Intracellular ROS were detected using a ROS assay kit (Genmed Scientifics Inc., Shanghai, China) containing an oxidation-sensitive fluorescent probe (DCFH-DA) with a spectrofluorometer (excitation 490 nm, emission 520 nm). Malondialdehyde (MDA) is an end-product of ROS-induced peroxidation. Superoxide Dismutase (SOD) is an important enzyme that participates in the removal of ROS from the cellular environment. The extent of lipid peroxidation was estimated by MDA levels, which were measured by using the spectrophotometric diagnostic kits (Jiancheng Biological Technology Co., Ltd., Nanjing, China) according to the manufacturer’s instructions. The SOD activity was determined using a SOD assay kit (Jiangsu KeyGEN BioTECH Co., Ltd., Nanjing, China) according to manufacturer’s instructions. Enzyme activity was converted to units per milligram of protein. One unit of SOD activity was defined as the amount that reduced the absorbance at 550 nm by 50%.

### Western Blotting Analysis

Hippocampal homogenate was prepared for the determination of SOD2 expression by standard western blotting. Total protein (35 μg/lane) was electrophoretically separated and blotted onto nitrocellulose membrane. Protein levels were determined via incubation with antibodies against SOD2 (1:1000; Abcam, Cambridge, MA, USA) and tubulin (1:1000; Sigma, St. Louis, MO, USA). Bands were visualized by enhanced chemiluminescence and quantified with the Image Quant Software (Syngene).

### Determination of Mitochondrial Swelling

Hippocampal tissue was removed immediately post-anesthesia and cut into small pieces for mitochondria isolation. The tissue was homogenized in 1.5 ml lysis buffer supplemented with protease inhibitor solution (QIAGEN China Co., Ltd., Shanghai, China) and centrifuged at 1000 g for 10 min at 4°C. The cell pellet was resuspended and homogenized in 1.5 ml ice-cold disruption buffer and centrifuged at 1000 g for 10 min. The supernatant was carefully transferred to a clean 1.5 ml tube and centrifuged at 6000 g for 10 min. The mitochondrion-enriched pellet was resuspended in 1 ml mitochondrial storage buffer and centrifuged at 6000 g for 20 min at 4°C.

The hippocampal mitochondrial swelling assay was performed by measuring the changes of the absorbance following addition of the mitochondrial suspension at 540 nm for 10 min using a colorimetric assay kit (Genmed Scientifics Inc., Wilmington, DE, USA). A decrease in absorbance represents the degree of mitochondrial swelling.

### Electron Microscopy

At PND 21, rats (*n* = 6 for each group) were perfused with normal saline, followed by 4% paraformaldehyde. Brains were then immersed in 4% paraformaldehyde for later embedding. Ultrastructural changes in hippocampal mitochondria were assessed by transmission electron microscopy. Briefly, the brain was fixed with 4% buffered glutaraldehyde and postfixed with 1% osmium tetroxide. The preparation was dehydrated through an ethanol gradient, processed for Epon 812 embedding, and sectioned at a thickness of 80 nm on a rotary microtome. The ultrathin sections were stained with 4% uranyl acetate-lead citrate and examined with a Tecnai G2 Transmission Electron Microscopy (FEI Company, Hillsboro, OR, USA). Electron microscope photographs were analyzed using Image-Pro Plus 6.1 software (Media Cybernetics, Silver Spring, MD, USA). We analyzed four neurons from each rat (*n* = 6 rats/group) for a total of 24 neurons in each group. Morphometric analyses were conducted as previously described by an investigator blinded to the experimental conditions (Sanchez et al., 2011; Boscolo et al., 2012, 2013a).

### Immunohistochemical Analysis

Paraffin sections were deparaffinized and hydrated using the following incubation steps: 10 min in xylene twice; 5 min in 100%, 10 min in 95%, 10 min in 85%, and 10 min in 70% ethanol; and 5 min three times in PBS at room temperature. Antigen retrieval was achieved by boiling the sections in 10 mM sodium citrate for 10 min in a microwave oven. The sections were washed with PBS three times, and treated with 3% H_2_O_2_-methanol for 15 min. Immunostaining was performed by incubation with antibody against cleaved caspase 3 (1:200; Cell Signaling Technology, Beverly, MA, USA) for 2 h. Sections were then washed three times and incubated with secondary antibodies labeled with horseradish peroxidase for 30 min at room temperature. Immunohistochemical (IHC) imaging was performed using ZEISS HB050 (Zeiss, Jena, Germany) inverted microscope system and handled by Image-Pro Plus 6.1 software (Media Cybernetics, Rockville, MD, USA). Cells with brownish-yellow cytoplasm were counted as positive cells. The numbers of caspase 3 immunoreactive cells in the hippocampal CA1 and dentate gyrus (DG) regions were counted and analyzed in four microscopic fields (at ×200 magnification) by an investigator blinded to the treatment conditions.

### TUNEL Fluorescent Assays

The TUNEL assay was carried out with the *in situ* Cell Death Detection Kit (Roche Inc., Indianapolis, IN, USA) following the protocols as previously described (Wu et al., 2015a). Sections were counterstained by 4′,6-diamidino-2-phenylindole (DAPI, Beyotime Institute of Biotechnology, Shanghai, China) for 3 min, washed with PBS three times, and covered by microscopic glass with antifade mounting medium (Beyotime) for further analyses. Fluorescence microscopy imaging was performed using ZEISS HB050 (Zeiss, Jena, Germany) inverted microscope system and handled by Image-Pro Plus 6.1 software (Media Cybernetics, Rockville, MD, USA). The positive cells were identified, counted and analyzed by an investigator blinded to the grouping. The apoptotic index was defined as the average percentage of TUNEL-positive cells in each section counted in four hippocampal CA1 microscopic fields (at ×400 magnification).

### Open Field Tests

To evaluate the anxiety behavior and general locomotor activity, we subjected rats (*n* = 12 for each group) to the open field test at PND 40. Each rat was gently placed in the center of a black plastic chamber (100 cm × 100 cm × 40 cm) for 10 min, while exploratory behavior was automatically recorded by a video tracking system (XR-XZ301, Shanghai Soft Maze Information Technology Co., Ltd., Shanghai, China). The total distance and the amount of time traveled in the center area (50 cm × 50 cm) of the maze were measured. After each test, the arena was cleaned with 75% alcohol to avoid the presence of olfactory cues.

### Morris Water Maze (MWM) Tests

To investigate spatial learning and memory function, we subjected rats (*n* = 12 for each group) to the Morris water maze (MWM) test (XR-XM101; Shanghai Xinruan Information Technology Co., Ltd., Shanghai, China) at PND 60. The MWM was a black metal tank (120 cm in diameter, 60 cm in depth) equipped with a platform (10 cm in diameter) 1–2 cm below the surface of the water. The MWM task was performed according to our previous study (Zhang et al., 2016). Briefly, it consisted of two phases, training phase for five consecutive days and probe trial phase on day 6. In the training phase, the rat was allowed to face to the pool wall in four random places (N, S, E, W) in the pool to find the fixed platform. Release positions were randomly predetermined. The trial was terminated once the rat reached the platform. If the rat failed to reach the platform within 60 s, it would be guided to the platform and allowed to stay for 15 s, then the latency was recorded for 60 s. In the probe test, single-probe trial was conducted with the original platform removed 24 h after the last training session. The rat was released at a random start position and allowed to swim for 60 s in the pool.

### Statistical Analysis

Data are presented as the mean ± SEM and analyzed by the Statistical Product for Social Sciences (SPSS; version 17.0, Chicago, IL, USA). The difference between the groups was determined by one-way analysis of variance followed by the Tukey’s tests. Comparisons for the spatial training sessions of MWM and mitochondrial size classification were performed by repeated two-way analysis of variance (ANOVA) followed by LSD test. A *p* value < 0.05 was regarded as statistical significance.

## Results

### Elamipretide Inhibits ROS Accumulation and Oxidative Stress in the Hippocampus of Developing Rats Exposed to Isoflurane

**Figure 1:**
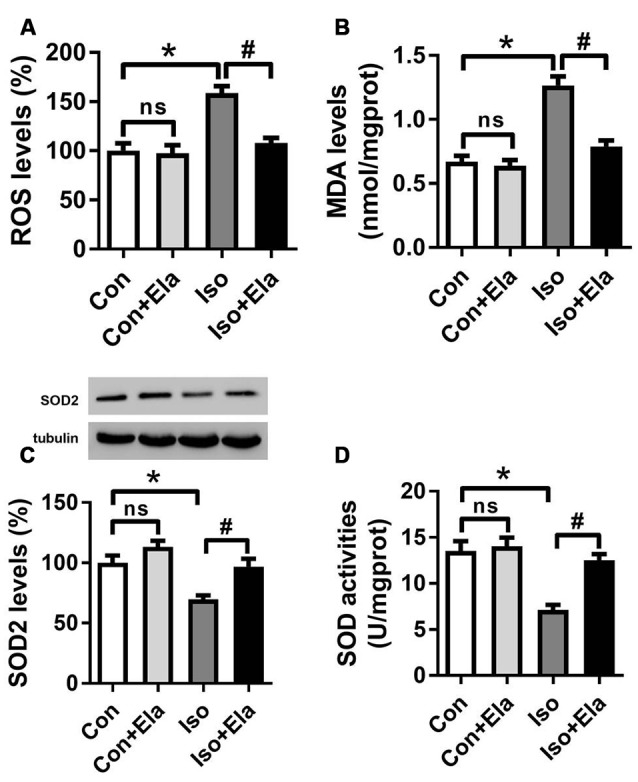
**Effects of elamipretide pretreatment on isoflurane-induced oxidative stress in the hippocampus of developing rats.** Elamipretide (5 mg/kg) or phosphate-buffered saline (PBS) was intraperitoneally administered to the pups with a volume of 0.4 ml/kg 30 min before gas inhalation. Anesthesia was induced by placing the pups in an anesthetizing chamber prefilled with 1.8% isoflurane plus 30% oxygen (O_2_) for 10 min and then changed to 1.5% isoflurane plus 30% O_2_ for 350 min. For control experiments, 30% O_2_ was delivered for 6 h at the same flow rate. Con: rats treated with PBS and 30% O_2_; Con + Ela: rats treated with elamipretide and 30% O_2_; Iso: rats treated with PBS and isoflurane; Iso + Ela: rats treated with elamipretide and isoflurane. The definition of Con/Con + Ela/Iso/Iso + Ela is the same as in the following figures. Reactive oxygen species (ROS) levels **(A)**, Malondialdehyde (MDA) levels **(B)**, Superoxide Dismutase (SOD) protein levels **(C)** and SOD activities **(D)** were determined with the fresh homogenates of hippocampal tissues obtained from postnatal day (PND) 7 rats (See “Materials and Methods” Section) immediately after 6 h of gas inhalation. Values are presented as mean ± SEM (*n* = 6 rats/group). *p < 0.05 vs. the control group; ^#^*p* < 0.05 vs. the isoflurane group; ns, no significance.

Since early exposure to isoflurane would induce excessive ROS accumulation inside the mitochondria, leading to the subsequent oxidative stress (Zhang et al., 2010; Boscolo et al., 2012, 2013a; Sun et al., 2014), we initially examined whether mitochondrion-targeted antioxidant elamipretide could timely inhibit hippocampal ROS accumulation and oxidative stress induced by isoflurane exposure. Our results showed that the levels of ROS (Figure 1A) and MDA (Figure 1B) were increased, while the expression and activity of SOD were reduced (Figures 1C,D) in the isoflurane group. However, the consequence was curtailed by elamipretide pretreatment (Figure 1), indicating that elamipretide provided rapidly antioxidative effects by inhibiting ROS accumulation and oxidative stress in the hippocampus of developing rat brain exposed to isoflurane.

### Elamipretide Reverses Mitochondrial Ultrastructural Damages in the Hippocampus of Developing Rats Exposed to Isoflurane

**Figure 2:**
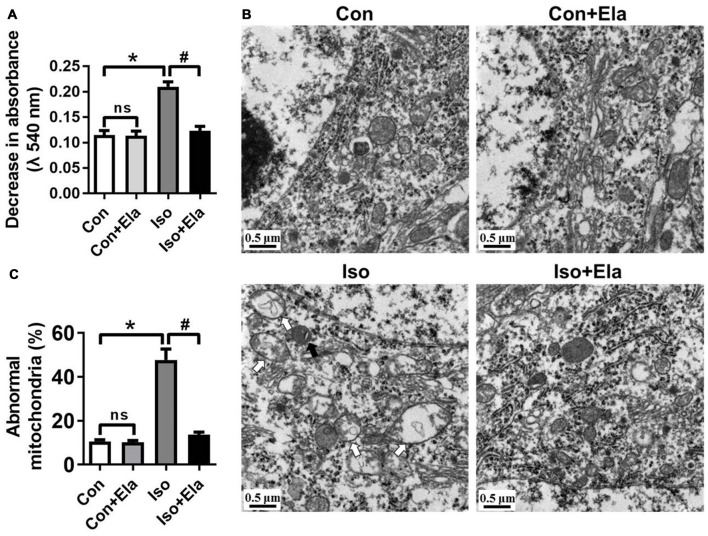
**Ultrastructural changes in mitochondria of the hippocampal neurons after elamipretide pretreatment and isoflurane anesthesia. (A)** Mitochondrial swelling was measured from fresh homogenates of the hippocampal tissues obtained from PND 7 rats. **(B)** Representative images of mitochondrial ultrastructure in hippocampal neuron of the developing rats at PND 21. White arrows: swollen mitochondria with disintegrated and disorganized mitochondrial inner membrane. Black arrows: degenerative mitochondria with dark and condensed presence. *Scale bar* = 0.5 μm for all the photographs. **(C)** Quantification analysis of abnormal-looking mitochondria in hippocampus of each group. Values are presented as mean ± SEM (*n* = 6 rats/group, four neurons from each rat). *p < 0.05, vs. the control group; ^#^*p* < 0.05 vs. the isoflurane group; ns, no significance.

**Figure 3:**
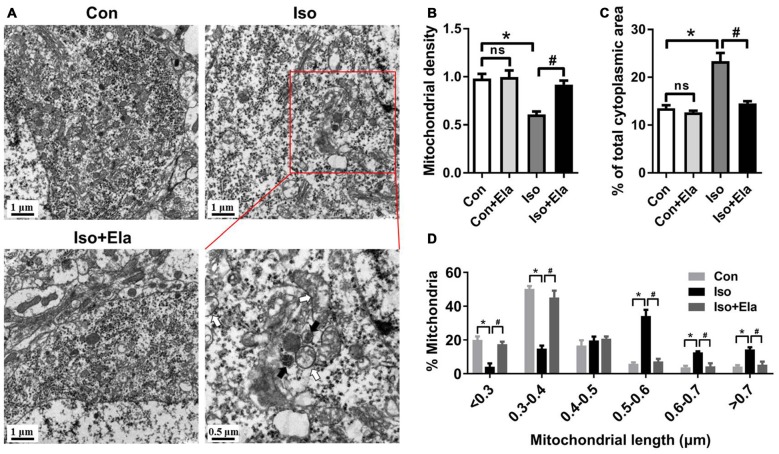
**Mitochondrial density and size classification in the hippocampal neurons after elamipretide pretreatment and isoflurane anesthesia. (A)** Mitochondrial overview in hippocampal neuron of the developing rats at PND 21. *Scale bar* = 1 μm for all the photographs except for the last one (*scale bar* = 0.5 μm). **(B)** Mitochondrial density was assessed by counting the number of mitochondria per unit area (μm^2^) of cytoplasmic area in each neuron. **(C)** Mitochondrial area was presented as a percentage of the cytoplasmic area of hippocampal neuron. **(D)** Size classification was assessed by mitochondrial size and counting the number of mitochondria in each group. Values are presented as mean ± SEM (*n* = 6 rats/group, four neurons from each rat). *p < 0.05, vs. the control group; ^#^*p* < 0.05 vs. the isoflurane group; ns, no significance.

Early exposure to GA may cause oxidative damages and long-term disturbances of mitochondrial morphology in developing rat brain (Sanchez et al., 2011; Boscolo et al., 2012). Here, we assessed whether timely diminishing substantial ROS and attenuating oxidative stress would ameliorate the damages of mitochondrial ultrastructure in developing rats exposed to isoflurane. We noted that isoflurane exposure caused early mitochondrial swelling (Figure 2A) at PND 7, and subsequently ultrastructural abnormalities (Figures 2B,C) at PND 21. In the isoflurane group, many mitochondria presented disorganized and vacuolated cristae (white arrows, Figures 2B, 3A) which made the mitochondria appear over-swelling and larger than normal ones. Other mitochondria were dark and condensed, indicating severe degenerative changes (black arrows, Figures 2B, 3A). In contrast, mitochondrial ultrastructure in the elamipretide-treated isoflurane group appeared similar to the control group with normal morphology of mitochondria without swelling or injury (Figures 2B,C). These results demonstrated that treatment with elamipretide before isoflurane exposure offered complete and lasting protection against isoflurane-induced ultrastructural damages of mitochondria in the hippocampus of developing rats.

### Elamipretide Rescues Changes of Mitochondrial Density and Size in the Hippocampus of Developing Rats Exposed to Isoflurane

Since GA appears to cause mitochondrial enlargement and disturb the regional distribution of developing neuronal mitochondria (Sanchez et al., 2011; Boscolo et al., 2012, 2013a), we performed detailed morphometric analysis of each mitochondrion and determined mitochondrial density in the soma of hippocampal neurons. We calculated mitochondrial density by counting the number of mitochondria per unit area (μm^2^) of cytoplasmic soma and measured mitochondrial areas as a percentage of the cytoplasmic area in each neuron (Figures 3A–C). The results showed that mitochondrial density in isoflurane group is less than that in control group (Figures 3A,B), but the mitochondria occupied more area of the cytoplasmic soma area in the isoflurane group than in the control group (Figures 3A,C). When classing mitochondria by their sizes and counting the number of mitochondria in each cell, we found more mitochondria larger than 0.5 μm in the isoflurane group than in the control group (Figure 3D). Interestingly, all the changes were completely reversed by elamipretide pretreatment (Figure 3). These results suggest that elamipretide offers mitochondrial protective effects associated with maintenance of mitochondrial density and size in spite of isoflurane exposure.

### Elamipretide Suppresses Neuroapoptosis in the Hippocampus of Developing Rats Exposed to Isoflurane

**Figure 4:**
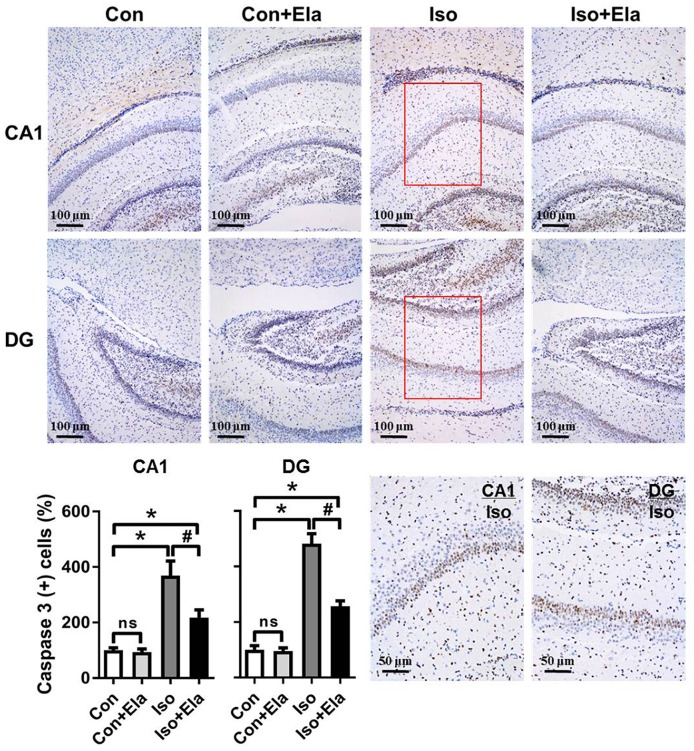
**Isoflurane-induced activation of caspase 3 was attenuated by elamipretide pretreatment in the hippocampal CA1 and dentate gyrus (DG) regions.** Representative images of cleaved caspase 3 in the hippocampal CA1 and DG regions are shown, revealed by immunohistochemical (IHC) staining at PND 21. Cells with brownish-yellow cytoplasm are positive for cleaved caspase 3. *Scale bar* = 100 μm for all the photographs except for the last two (*scale bar* = 50 μm). Lower panel presents statistical data from the four experimental groups. Values are presented as mean ± SEM (*n* = 6 rats/group). *p < 0.05, vs. the control group; ^#^*p* < 0.05 vs. the isoflurane group; ns, no significance.

**Figure 5:**
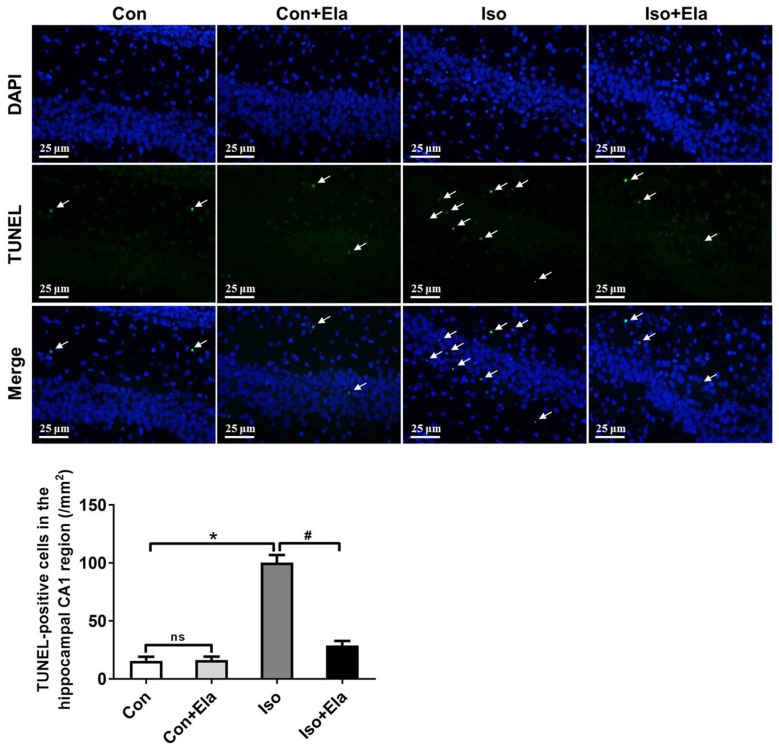
**The number of TUNEL-positive cells was diminished by elamipretide pretreatment in the hippocampal CA1.** Representative images of TUNEL in the hippocampal CA1 region at PND 21 are shown. Green color indicates TUNEL-positive cells; blue, 4′,6-diamidino-2-phenylindole (DAPI) stained nucleus. *Scale bar* = 25 μm. The lower panel shows statistical numbers of TUNEL-positive cells. Values are presented as mean ± SEM (*n* = 6 rats/group). *p < 0.05, vs. the control group; ^#^*p* < 0.05 vs. the isoflurane group; ns, no significance.

Because isoflurane not only caused severe ROS generation and oxidative stress, but also disturbed mitochondrial morphogenesis, we examined whether these effects had long-lasting consequence with regard to developmental neuroapoptosis and whether elamipretide pretreatment would attenuate this neurotoxicity. Our results showed that prolonged (6 h) isoflurane exposure induced significant increase of caspase 3 (Figure 4) and the number of TUNEL-positive cells (Figure 5) in hippocampus, which was attenuated by elamipretide pretreatment, supporting that mitochondrion-targeted peptide elamipretide presented a long-lasting protection of hippocampal neurons from apoptosis.

### Elamipretide Ameliorates Isoflurane-induced Long-term Cognitive Deficits in Adolescent Rats

**Figure 6:**
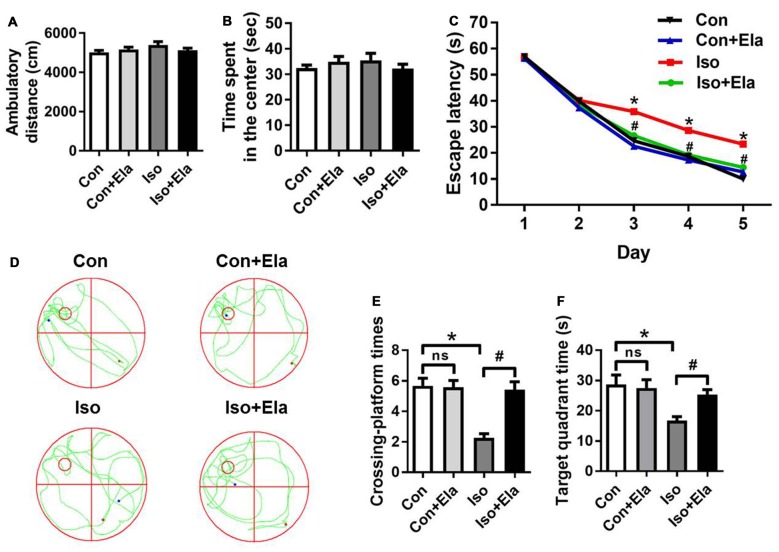
**Elamipretide pretreatment prevented isoflurane-induced cognitive deficits in adolescent rats, tested at PND 40 or 60. (A)** Total distance traveled and **(B)** time spent in the center in open field test. **(C)** Escape latency in every day during the spatial training of MWM. **(D)** Representative swimming trajectory of the rats, **(E)** crossing-platform times and **(F)** time spent in the target quadrant in the probe trial of MWM. Values are presented as mean ± SEM (*n* = 12 rats/group). *p < 0.05 vs. the control group; ^#^*p* < 0.05 vs. the isoflurane group; ns, no significance. The procedures of the water maze test are in “Materials and Methods” Section. MWM, Morris water maze.

To further verify the beneficial effects of elamipretide on cognitive development, open field and MWM tests were performed at PND 40 or 60, respectively. Open field test showed no difference among the four groups in the spontaneous locomotor activity as reflected by the total distance (Figure 6A) and the time spent in the center (Figure 6B), excluding the possibility that locomotor activity *per se* affected the results in MWM tests. Elamipretide pretreatment successfully shortened the escape latency in training test (Figure 6C) and increased the crossing-platform times (Figures 6D,E) and target quadrant time (Figures 6D,F) in probe trial in the developing rats exposed to isoflurane. These results suggest that elamipretide has a potential therapeutic effect on long-term cognitive deficits in adolescent rats after early exposure to isoflurane.

## Discussion

In the present study, we showed that early exposure to isoflurane for the developing rats caused increased oxidative stress, mitochondrial deformation and neuronal apoptotic toxicity in the hippocampus. Pretreatment with mitochondrion-targeted antioxidant elamipretide resulted in significant improvement of mitochondrial integrity in the early and later period and, most importantly, prevented the young rats from development of anesthesia-induced cognitive deficits.

A growing body of preclinical as well as some retrospective clinical evidences suggests that exposure to GA during the critical periods of development has been associated with neurotoxicity and long-term behavioral impairments (Flick et al., 2011; Ing et al., 2014b; Lin et al., 2014; Rappaport et al., 2015; Pinyavat et al., 2016; Walters and Paule, 2017). Consistently, we found that isoflurane administration induced long lasting neuronal apoptosis and cognitive deficits in developing rats. Mitochondria are supposed to be the most vulnerable initial target of these disorders (Sanchez et al., 2011; Boscolo et al., 2012, 2013a,b). Available evidences accompanying with our results suggest that rats exposed to isoflurane at PND 7 present substantial alterations in mitochondrial morphogenesis and regional distribution, manifested as a decrease of mitochondrial size as acute effects (at PND 8; Boscolo et al., 2013a) and an enlargement of mitochondria as long-term effects (at PND 21; Sanchez et al., 2011; Boscolo et al., 2012). Our findings are in agreement with previous studies (Sanchez et al., 2011; Boscolo et al., 2012) demonstrating that prolonged (6 h) isoflurane exposure causes swollen mitochondria with lower density and more cytoplasmic area occupation in the soma of hippocampal neurons at PND 21, suggesting long-term ultrastructural damages of mitochondria. It is likely that the swelling enlargement leads to “leakage” and results in neurotoxicity via activating the intrinsic mitochondria-dependent apoptotic pathway (Zhang et al., 2010; Wu et al., 2015a).

Agents and strategies have been proposed to be potential to ameliorate the mitochondrial morphologic abnormality induced by neonatal GA (Boscolo et al., 2012, 2013a; Xu et al., 2016). These findings suggest that mitochondrial ROS as well as lipid peroxidation are important in the development and progression of impaired mitochondrial morphogenesis. In agreement with these, molecules that are capable of targeting and treating the dysfunctional mitochondria to suppress oxidative stress are needed. Elamipretide is a novel mitochondrion-targeted peptide to meet the need (Zhao et al., 2005; Szeto, 2008; Min et al., 2011; Hao et al., 2015; Yin et al., 2016). Previously, we found that elamipretide prevented isoflurane-induced mitochondrial ROS generation to improve mitochondrial function in aged mice (Wu et al., 2015a, 2016). Here, we show that elamipretide attenuates acute ROS accumulation induced by isoflurane exposure and, more importantly, protects mitochondrial morphology from enlargement in neonatal rats lasting to early adulthood. As a result, elamipretide ameliorated isoflurane-induced long-term cognitive deficits. Thus, we provide the evidences that elamipretide not only improves biochemical and pathological processes, but also the preclinical symptoms. Consistence with that, elamipretide was reported to reduce impaired mitochondrial dynamics and enhance mitochondrial biogenesis in Huntington’s disease neurons (Yin et al., 2016). Due to the superpower properties of elamipretide, more than 20 clinical trials (registered as elamipretide or Bendavia) have been being conducted as phase 1 or 2 drug^1^ covering a number of mitochondrial disorders. So far the safety and tolerability were well confirmed (Gibson et al., 2016), which might potentially raise a new therapeutic strategy for elamipretide as a drug to protect pediatric patients undertaking GA.

In conclusion, this is the first study to our knowledge that directly targeting mitochondrial ROS ameliorated isoflurane-induced long-term impairments of mitochondrial morphogenesis and cognition in developing rats. This work suggests a new strategy to reverse developing- and/or mitochondrion-related impairments in GA-induced neurotoxicity and cognitive deficits.

## Author Contributions

JW, KL and J-JY conceived and designed experiments. JW and SH performed experiments and data acquisition. JW, SH, KL and J-JY analyzed and interpreted data. JW and KL wrote the manuscript. X-RS, HZ, HL, HtZ and M-HJ contributed to acquiring and analyzing data. All authors read and approved the final manuscript.

## Conflict of Interest Statement

The authors declare that the research was conducted in the absence of any commercial or financial relationships that could be construed as a potential conflict of interest.

## Footnotes

^1^https://clinicaltrials.gov/

## References

[B1] Boscolo A. Milanovic D. Starr J. A. Sanchez V. Oklopcic A. Moy L.. (2013a). Early exposure to general anesthesia disturbs mitochondrial fission and fusion in the developing rat brain. Anesthesiology 118, 1086–1097. 10.1097/ALN.0b013e318289bc9b 23411726 PMC3879793

[B2] Boscolo A. Ori C. Bennett J. Wiltgen B. Jevtovic-Todorovic V. (2013b). Mitochondrial protectant pramipexole prevents sex-specific long-term cognitive impairment from early anaesthesia exposure in rats. Br. J. Anaesth. 110, i47–i52. 10.1093/bja/aet073 23616588 PMC3732064

[B3] Boscolo A. Starr J. Sanchez V. Lunardi N. DiGruccio M. Ori C.. (2012). The abolishment of anesthesia-induced cognitive impairment by timely protection of mitochondria in the developing rat brain: the importance of free oxygen radicals and mitochondrial integrity. Neurobiol. Dis. 45, 1031–1041. 10.1016/j.nbd.2011.12.022 22198380 PMC3276740

[B4] Flick R. P. Katusic S. K. Colligan R. C. Wilder R. T. Voigt R. G. Olson M. D.. (2011). Cognitive and behavioral outcomes after early exposure to anesthesia and surgery. Pediatrics 128, e1053–e1061. 10.1542/peds.2011-0351 21969289 PMC3307194

[B5] Gibson C. M. Giugliano R. P. Kloner R. A. Bode C. Tendera M. Janosi A.. (2016). EMBRACE STEMI study: a Phase 2a trial to evaluate the safety, tolerability, and efficacy of intravenous MTP-131 on reperfusion injury in patients undergoing primary percutaneous coronary intervention. Eur. Heart J. 37, 1296–1303. 10.1093/eurheartj/ehv597 26586786

[B6] Hao S. Ji J. Zhao H. Shang L. Wu J. Li H.. (2015). Mitochondrion-targeted peptide SS-31 inhibited oxidized low-density lipoproteins-induced foam cell formation through both ROS scavenging and inhibition of cholesterol influx in RAW264.7 cells. Molecules 20, 21287–21297. 10.3390/molecules201219764 26633327 PMC6332157

[B7] Ing C. H. DiMaggio C. J. Malacova E. Whitehouse A. J. Hegarty M. K. Feng T.. (2014a). Comparative analysis of outcome measures used in examining neurodevelopmental effects of early childhood anesthesia exposure. Anesthesiology 120, 1319–1332. 10.1097/ALN.0000000000000248 24694922

[B8] Ing C. H. DiMaggio C. J. Whitehouse A. J. Hegarty M. K. Sun M. von Ungern-Sternberg B. S.. (2014b). Neurodevelopmental outcomes after initial childhood anesthetic exposure between ages 3 and 10 years. J. Neurosurg. Anesthesiol. 26, 377–386. 10.1097/ANA.0000000000000121 25144506

[B9] Jevtovic-Todorovic V. Boscolo A. Sanchez V. Lunardi N. (2012). Anesthesia-induced developmental neurodegeneration: the role of neuronal organelles. Front. Neurol. 3:141. 10.3389/fneur.2012.00141 23087668 PMC3468830

[B10] Lin E. P. Soriano S. G. Loepke A. W. (2014). Anesthetic neurotoxicity. Anesthesiol. Clin. 32, 133–155. 10.1016/j.anclin.2013.10.003 24491654

[B11] Min K. Smuder A. J. Kwon O. S. Kavazis A. N. Szeto H. H. Powers S. K. (2011). Mitochondrial-targeted antioxidants protect skeletal muscle against immobilization-induced muscle atrophy. J. Appl. Physiol. 111, 1459–1466. 10.1152/japplphysiol.00591.2011 21817113 PMC3220313

[B12] Noguchi K. K. Johnson S. A. Kristich L. E. Martin L. D. Dissen G. A. Olsen E. A.. (2016). Lithium protects against anaesthesia neurotoxicity in the infant primate brain. Sci. Rep. 6:22427. 10.1038/srep22427 26951756 PMC4782073

[B13] Pinyavat T. Warner D. O. Flick R. P. McCann M. E. Andropoulos D. B. Hu D.. (2016). Summary of the update session on clinical neurotoxicity studies. J. Neurosurg. Anesthesiol. 28, 356–360. 10.1097/ANA.0000000000000347 27768673 PMC5077165

[B14] Rappaport B. A. Suresh S. Hertz S. Evers A. S. Orser B. A. (2015). Anesthetic neurotoxicity—clinical implications of animal models. N. Engl. J. Med. 372, 796–797. 10.1056/NEJMp1414786 25714157

[B15] Sanchez V. Feinstein S. D. Lunardi N. Joksovic P. M. Boscolo A. Todorovic S. M.. (2011). General anesthesia causes long-term impairment of mitochondrial morphogenesis and synaptic transmission in developing rat brain. Anesthesiology 115, 992–1002. 10.1097/ALN.0b013e3182303a63 21909020 PMC3203321

[B16] Sun Y. Cheng B. Dong Y. Li T. Xie Z. Zhang Y. (2014). Time-dependent effects of anesthetic isoflurane on reactive oxygen species levels in HEK-293 cells. Brain Sci. 4, 311–320. 10.3390/brainsci4020311 24961763 PMC4101479

[B17] Szeto H. H. (2008). Mitochondria-targeted cytoprotective peptides for ischemia-reperfusion injury. Antioxid. Redox Signal. 10, 601–619. 10.1089/ars.2007.1892 17999629

[B18] Walters J. L. Paule M. G. (2017). Review of preclinical studies on pediatric general anesthesia-induced developmental neurotoxicity. Neurotoxicol. Teratol. 60, 2–23. 10.1016/j.ntt.2016.11.005 27871903

[B19] Wang W. Y. Luo Y. Jia L. J. Hu S. F. Lou X. K. Shen S. L.. (2014). Inhibition of aberrant cyclin-dependent kinase 5 activity attenuates isoflurane neurotoxicity in the developing brain. Neuropharmacology 77, 90–99. 10.1016/j.neuropharm.2013.09.006 24055498

[B20] Wu J. Li H. Sun X. Zhang H. Hao S. Ji M.. (2015a). A mitochondrion-targeted antioxidant ameliorates isoflurane-induced cognitive deficits in aging mice. PLoS One 10:e0138256. 10.1371/journal.pone.0138256 26379247 PMC4575031

[B21] Wu J. Zhang M. Hao S. Jia M. Ji M. Qiu L.. (2015b). Mitochondria-targeted peptide reverses mitochondrial dysfunction and cognitive deficits in sepsis-associated encephalopathy. Mol. Neurobiol. 52, 783–791. 10.1007/s12035-014-8918-z 25288156

[B22] Wu J. Zhang M. Li H. Sun X. Hao S. Ji M.. (2016). BDNF pathway is involved in the protective effects of SS-31 on isoflurane-induced cognitive deficits in aging mice. Behav. Brain Res. 305, 115–121. 10.1016/j.bbr.2016.02.036 26944333

[B23] Xu F. Armstrong R. Urrego D. Qazzaz M. Pehar M. Armstrong J. N.. (2016). The mitochondrial division inhibitor Mdivi-1 rescues mammalian neurons from anesthetic-induced cytotoxicity. Mol. Brain 9:35. 10.1186/s13041-016-0210-x 27009068 PMC4806411

[B24] Xu K. X. Tao J. Zhang N. Wang J. Z. (2015). Neuroprotective properties of vitamin C on equipotent anesthetic concentrations of desflurane, isoflurane, or sevoflurane in high fat diet fed neonatal mice. Int. J. Clin. Exp. Med. 8, 10444–10458. 26379835 PMC4565218

[B25] Yin X. Manczak M. Reddy P. H. (2016). Mitochondria-targeted molecules MitoQ and SS31 reduce mutant huntingtin-induced mitochondrial toxicity and synaptic damage in Huntington’s disease. Hum. Mol. Genet. 25, 1739–1753. 10.1093/hmg/ddw045 26908605 PMC4986329

[B26] Zhang H. Sun X. R. Wang J. Zhang Z. Z. Zhao H. T. Li H. H.. (2016). Reactive oxygen species-mediated loss of phenotype of parvalbumin interneurons contributes to long-term cognitive impairments after repeated neonatal ketamine exposures. Neurotox. Res. 30, 593–605. 10.1007/s12640-016-9653-1 27443555

[B27] Zhang Y. Dong Y. Wu X. Lu Y. Xu Z. Knapp A.. (2010). The mitochondrial pathway of anesthetic isoflurane-induced apoptosis. J. Biol. Chem. 285, 4025–4037. 10.1074/jbc.M109.065664 20007710 PMC2823544

[B28] Zhao K. Luo G. Giannelli S. Szeto H. H. (2005). Mitochondria-targeted peptide prevents mitochondrial depolarization and apoptosis induced by tert-butyl hydroperoxide in neuronal cell lines. Biochem. Pharmacol. 70, 1796–1806. 10.1016/j.bcp.2005.08.022 16216225

